# Mendelian randomization analysis of the causal relationship between serum metabolites and thoracic aortic aneurysm

**DOI:** 10.1097/MD.0000000000039686

**Published:** 2024-09-13

**Authors:** Xiaoshan Tong, Yu Cui

**Affiliations:** a Department of Cardiac Surgery, The First Hospital of China Medical University, Shenyang, China; b Department of Cardiology, The First Hospital of China Medical University, Shenyang, China.

**Keywords:** causal relationship, Mendelian randomization, metabolites, thoracic aortic aneurysm

## Abstract

Thoracic aortic aneurysm (TAA) is associated with changes in the levels of metabolites; however, the exact causal relationships remain unclear. Identifying this complex relationship may provide new insights into the pathogenesis of TAA. We used genome-wide association studies to investigate the relationship between metabolites and TAA in this study. A total of 1400 serum metabolites were investigated for their potential causal effects on the risk of TAA. We performed bidirectional and 2-sample Mendelian randomization (MR) analysis using 5 MR tests: MR-Egger, weighted mode, weighted median, inverse variance weighted (IVW), and simple mode. We also performed sensitivity analysis to verify our findings, including heterogeneity analysis using IVW and MR-Egger tests and pleiotropy analysis using the MR-Egger test. Multiple metabolites were identified as having a causal effect on the risk of TAA, particularly those related to lipid metabolites; the top 2 risk factors identified using the IVW test were 3-carboxy-4-methyl-5-pentyl-2-furanpropionate (*P* = .019) and 5alpha-androstan-3alpha,17alpha-diol (*P* = .021), whereas the 2 top protective factors were 1-stearoyl-2-docosahexaenoyl-gpc (*P* = .023) and 1-oleoyl-2-docosahexaenoyl-GPC (*P* = .005). Sensitivity analysis verified the lack of heterogeneity (*P* = .499, .584, .232, and .624, respectively; IVW test) or pleiotropy (*P* = .621, .483, .598, and .916, respectively; Egger test). Our study provides new evidence of a causal relationship between metabolites and the risk of TAA, thus providing new insights into the pathogenesis of this disease. These findings suggest a promising approach for metabolite-based therapeutic interventions.

**Key points**

- Lipid metabolism disorder is found to be involved in the pathogenesis of thoracic aortic aneurysm (TAA) by Mendelian randomization genetic analysis.
- 3-Carboxy-4-methyl-5-pentyl-2-furanpropionate and 5alpha-androstan-3alpha,17alpha-diol are the important risk factors for the formation of TAA.

## 1. Introduction

Thoracic aortic aneurysm (TAA) is a vascular surgical disease caused by multiple factors that can lead to aortic diameter dilation exceeding 50%.^[1,2]^ Approximately 80% of all cases of TAA are secondary to atherosclerosis in hypertension, and the incidence rate is increasing annually.^[3–5]^ TAA has a slow onset and often presents with no specific symptoms in the early stages but can also manifest as chest pain, thus increasing the risk of misdiagnosis as heart disease.^[6]^ Effective therapeutic drugs for TAA have yet to be developed; consequently, current treatment predominantly relies on surgery.^[7–9]^ Therefore, it is crucial that we investigate the pathogenic mechanisms underlying TAA.

Over recent years, many studies have verified a close correlation between metabolic abnormalities and the occurrence of cardiovascular disease (CVD). Studies have reported a significant association between phenylacetylglutamine, a plasma metabolite, and CVD. This metabolite increases the ability to form blood clots, thereby increasing the probability of related adverse events such as apoplexy and myocardial infarction.^[10]^ Previous research by Li et al revealed that various plasma metabolites, particularly those involved in the metabolism of fatty and amino acids, are associated with a high risk of CVD.^[11,12]^ Research on the intestinal flora and its dependent metabolites has also shown that the levels of metabolites after entering the blood vessels are closely related to cardiovascular stability and that trimethylamine N-oxide may participate in the formation of atherosclerosis.^[13]^ A previous Takayasu investigation by Fan et al found that various bacterial communities can induce vascular involvement via the metabolism of glycerophospholipid, alanine, aspartic acid, and glutamate, thus leading to the Takayasu phenotype.^[14]^ However, the causal relationship between metabolites and TAA remains unclear and requires further in-depth analysis.

Mendelian randomization (MR) is a data analysis technique used in epidemiological studies to evaluate causal inferences. MR uses genetic variations that are strongly correlated with exposure factors as instrumental variables to evaluate the causal relationship between exposure factors and outcomes.^[15]^ Recently, MR has become a widely accepted technique for revealing the causal relationship between exposure factors and diseases.^[16–18]^ In the present study, we acquired information related to serum metabolites and TAA from public databases and used MR genetic analysis to elucidate the relationship between metabolites and the risk of TAA. We aimed to provide a detailed interpretation of the role of metabolites as a risk or protective factor in the pathogenesis of TAA.

## 2. Methods

### 2.1. Data extraction

Information relating to the study on serum metabolites is described in detail on a public website (https://www.freebioinfo.org/), along with operational analyses. Data arising from the genome-wide association studies on TAA was acquired from the ebi-a-GCST90027266 database (https://gwas.mrcieu.ac.uk/), which contained 19,646 samples with 22,889,272 single-nucleotide polymorphisms.

### 2.2. MR genetic analysis

For MR analysis, we applied 5 tests: MR-Egger, weighted mode, weighted median, inverse variance weighted (IVW), and simple mode.

### 2.3. Sensitivity analysis

#### 2.3.1. Heterogeneity analysis

Heterogeneity may exist in instrumental variables arising from different analytical platforms, experiments, and populations, which could influence the results of MR analysis. We conducted IVW and MR-Egger tests on our data to identify the evidence of heterogeneity (*P* < .05).

#### 2.3.2. Pleiotropy analysis

An instrumental variable is considered to exhibit pleiotropy if it affects the outcome via factors other than exposure. Pleiotropy can lead to the failure of the hypotheses of independence and exclusivity. In our research, we used the MR-Egger intercept test to verify variable pleiotropy and evaluate the robustness of our results (*P* < .05).

### 2.4. Analysis tools

All statistical analyses were performed using R version 4.2.0. For MR analysis, we used TwoSampleMR (version 0.5.7) and MR-PRESSO (version 1.0).

## 3. Results

### 3.1. The causal effect of serum metabolites on TAA

**Figure 1.**
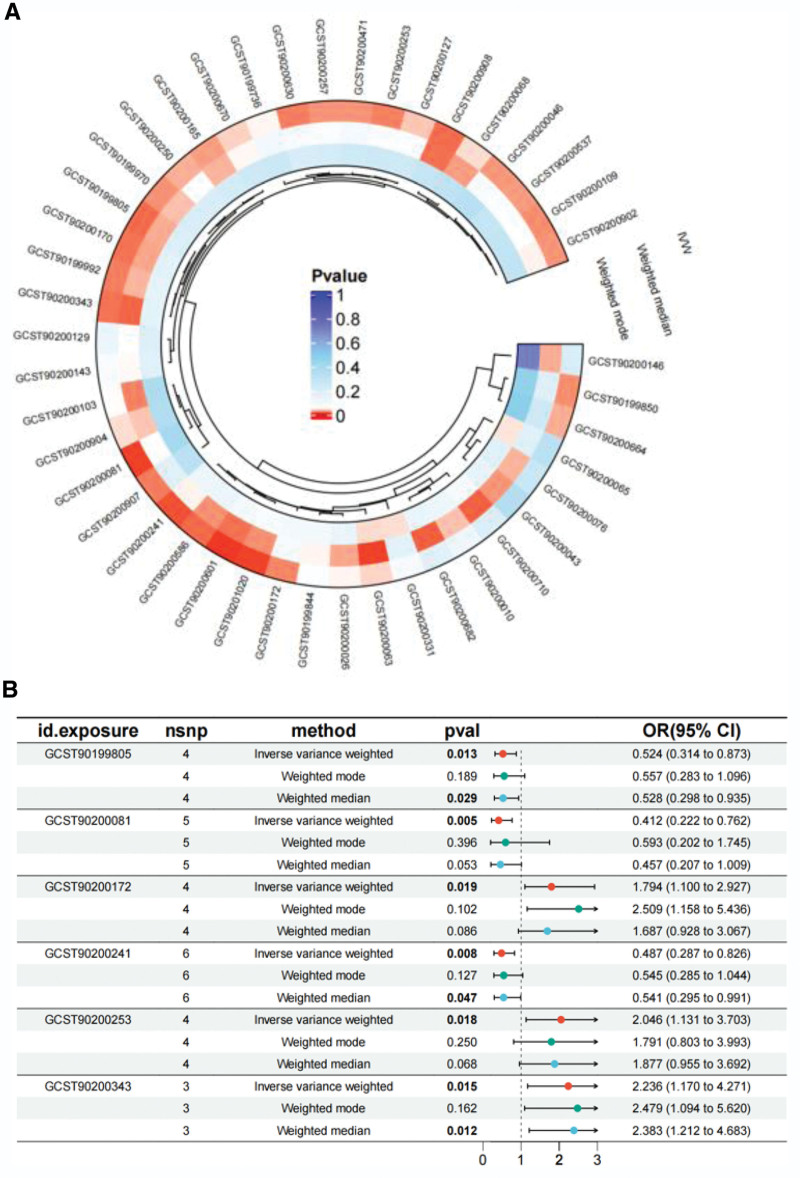
The circular heatmap (A) depicted a visualization of the directions of effect determined by the 3 MR methods (IVW, weighted media, and weighted mode). Random forest plot (B) showed the top 3 metabolites that exhibited a consistent direction of effect.

We used 3 different methods to investigate the causal relationship between serum metabolites and TAA. A circular heatmap was generated to depict the directions of effect determined by the 3 MR methods (IVW, weighted media, and weighted mode; Fig. 1A). Next, we generated a random forest plot to identify the top 3 metabolites that exhibited a consistent direction of effect (Fig. 1B). Most substances belong to lipid metabolites, thus indicating that lipid metabolism disorders are the main factors influencing the pathogenesis of TAA.

### 3.2. Identification of the top 2 risk and protective metabolites for TAA

**Figure 2.**
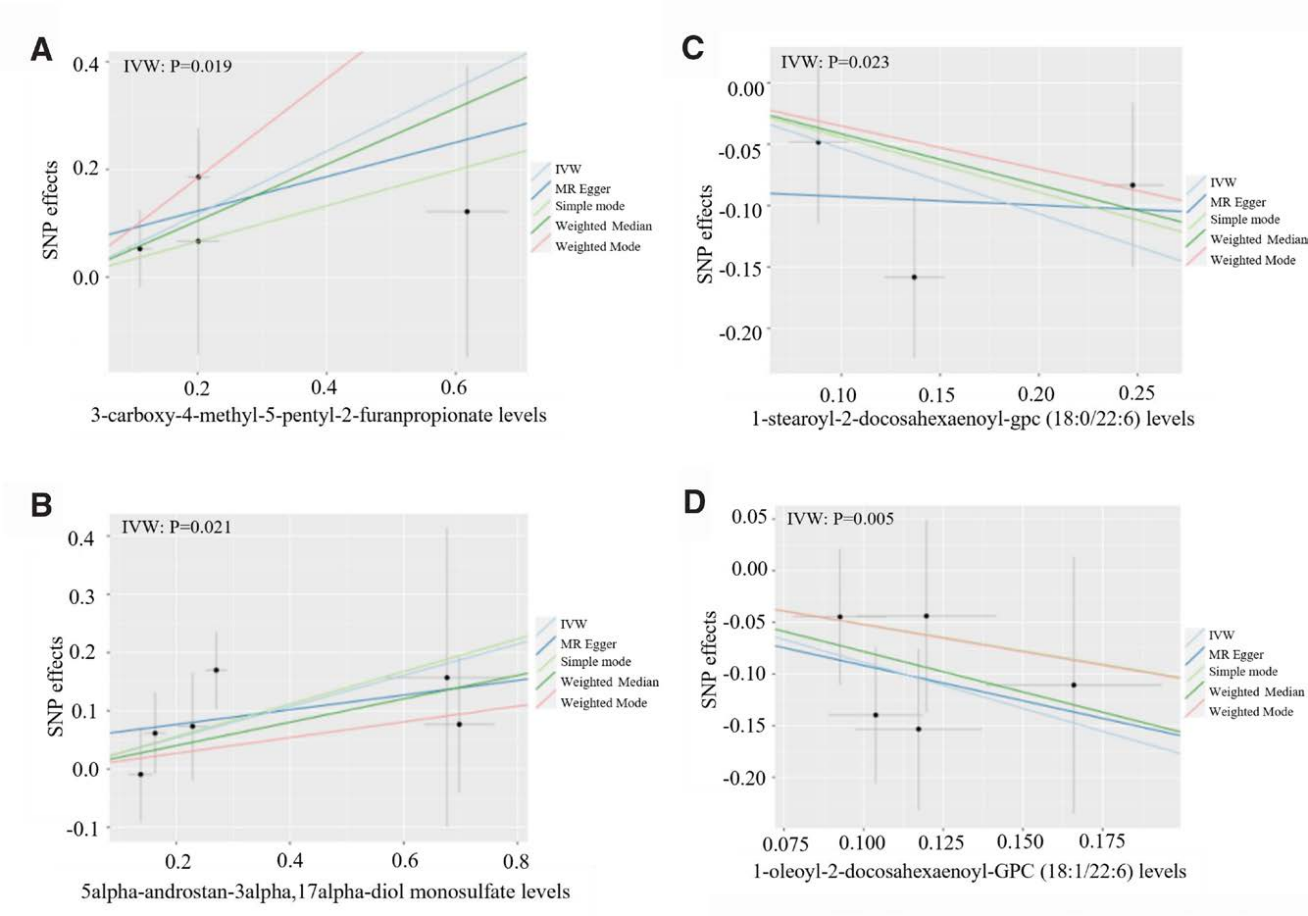
Scatterplot depicted the MR results for the top 2 ranked metabolites. IVW test identified 3-carboxy-4-methyl-5-pentyl-2-furanpropionate (*P* = .019, A) and 5alpha-androstan-3alpha,17alpha-diol (*P* = .021, B) are the top 2 risk factors, 1-stearoyl-2-docosahexaenoyl-gpc (*P* = .023, C) and 1-oleoyl-2-docosahexaenoyl-GPC (*P* = .005, D) are the top 2 protective factors.

Subsequently, we considered the lipid metabolites with the most consistent directions of effects and used 5 MR methods to identify the top-ranked metabolites. Figure 2 shows the MR results for the top 2 ranked metabolites; IVW test identified 3-carboxy-4-methyl-5-pentyl-2-furanpropionate (*P* = .019; Fig. 2A) and 5alpha-androstan-3alpha,17alpha-diol (*P* = .021; Fig. 2B) as the top 2 risk factors, and 1-stearoyl-2-docosahexaenoyl-gpc (*P* = .023; Fig. 2C) and 1-oleoyl-2-docosahexaenoyl-GPC (*P* = .005; Fig. 2D) as the top 2 protective factors for TAA. MR results for the other metabolites exhibiting causal relationships related to TAA are presented in Table S1, Supplemental Digital Content, http://links.lww.com/MD/N576 (which recorded MR results of differential metabolites exhibiting causal relationships related to TAA). However, further investigations are needed to identify specific biological effects.

### 3.3. Sensitivity analysis

**Figure 3.**
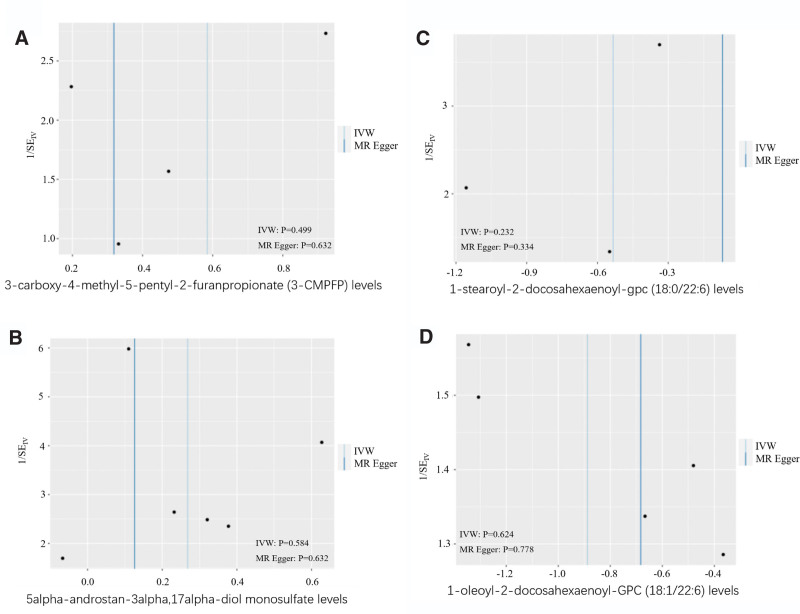
Heterogeneity analysis result of the 4 metabolites. IVW test generated *P* values of .499 (A), .584 (B), .232 (C), and .624 (D), respectively, which were consistent with those arising from Egger test, indicated that there was no evidence of heterogeneity.

**Figure 4.**
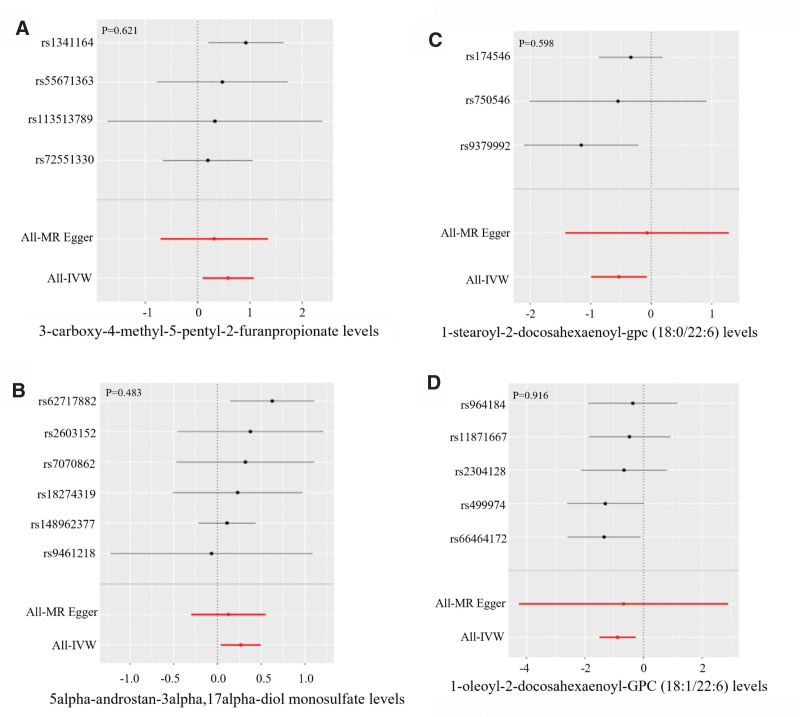
Pleiotropy analysis result of the 4 metabolites. MR-Egger test generated *P* values of .621 (A), .483 (B), .598 (C), and .916 (D), respectively, confirmed the lack of evidence of horizontal pleiotropy.

Next, we conducted IVW and MR-Egger tests to investigate for heterogeneity; the IVW test for the 4 metabolites generated *P* values of .499 (Fig. 3A), .584 (Fig. 3B), .232 (Fig. 3C), and .624 (Fig. 3D), respectively; these findings were consistent with those arising from Egger test, thus indicating that there was no evidence of heterogeneity. We also used the MR-Egger test to investigate pleiotropy; this test generated *P* values of .621 (Fig. 4A), .483 (Fig. 4B), .598 (Fig. 4C), .916 (Fig. 4D), respectively, thus confirming the lack of evidence of horizontal pleiotropy.

## 4. Discussion

This is the first study to apply MR genetic analysis and demonstrate that lipid metabolism disorders are associated with the occurrence and development of TAA. Specifically, we identified 3-carboxy-4-methyl-5-pentyl-2-furanpropionate and 5alpha-androstan-3alpha,17alpha-diol as the top 2 risk factors and 1-stearoyl-2-docosahexaenoyl-gpc and 1-oleoyl-2-docosahexaenoyl-GPC are the top 2 protective factors.

3-Carboxy-4-methyl-5-pentyl-2-furanpropionate is an endogenous furan fatty acid metabolite with a molecular weight of 240 kDa that was first discovered in urine and blood in the 1970s and was initially considered as a uremic toxin.^[19]^ Recently, Qiang Lai et al reported that 3-carboxy-4-methyl-5-pentyl-2-furanpropionate can damage heart function and exacerbate myocardial injury by regulating the processes involved in fatty acid oxidation.^[20]^ Other studies have demonstrated that 3-carboxy-4-methyl-5-pentyl-2-furanpropionate is involved in the induction of diabetes by directly acting on β cells, leading to impaired mitochondrial function and ultimately reducing the synthesis of insulin. This mechanism has already been validated in a population of Chinese individuals.^[21–23]^ These studies indicate that 3-carboxy-4-methyl-5-pentyl-2-furanpropionate can participate in the occurrence of diseases by regulating pathways responsible for lipid metabolism and by participating in the process of mitochondrial oxidative phosphorylation; these mechanisms are crucial pathogenic factors for TAA.^[24]^

5alpha-androstan-3alpha,17alpha-diol is a metabolite of dihydrotestosterone, plays a weak role in androgenesis, and can also act as a regulator to increase the expression of the prostate estrogen receptor Erβ.^[25]^ In a previous study, Zimmerman et al showed that 5alpha-androstan-3alpha,17alpha-diol, in combination with the androgen receptor pathway, can induce the EGF signaling pathway in prostate cancer to stimulate the proliferation of prostate cells and exacerbate the progression of disease.^[26]^ These findings suggested an association between the action of this metabolite on androgens and the formation of TAA. In addition, most previous research on 5alpha-androstan-3alpha,17alpha-diol has focused on neurodevelopment and mammalian mating and indicated that the excessive accumulation of this metabolite disturb mammalian neural growth, development, and reproduction ability.^[27]^ This functionality may be attributed to steady-state regulation of the endocrine system, which at the macro-level is known to be closely related to a variety of chronic diseases, including TAA.

1-Stearoyl-2-docosahexaenoyl-gpc is an important phospholipid in biofilms,^[28]^ acts as the main substrate for synthesizing acidic phospholipids, and participates in cellular signaling processes.^[29]^ Relatively, few previous studies have focused on 1-stearoyl-2-docosahexaenoyl-gpc; however, the role of phosphatidylethanolamines in this family is relatively clear. Some researchers have demonstrated that phosphatidylethanolamine reduces the accumulation and secretion of de novo lipogenesis and triglycerides in the liver by inhibiting the cleavage of sterol regulatory element-binding protein-1c, ultimately inhibiting the occurrence of fatty liver.^[30]^ In addition, phosphatidylethanolamine plays a protective role against diseases by inhibiting lipid peroxidation, which results in iron depletion and promotes autophagy.^[31]^

In the present study, we also identified that another phospholipid, 1-oleoyl-2-docosahexaenoyl-GPC, plays a similar role to 1-stearoyl-2-docosahexaenoyl-gpc. Higher levels of GPC (18:1/18:2) in patients with endometrial cancer have been shown to enhance membrane fluidity and promote the progression of endometrial cancer. Furthermore, GPC (18:1/18:2) was demonstrated to be an independent risk factor for lymph node metastasis positively associated with ERRα.^[32]^ In another study, Liu et al reported that GPC is a key metabolite that regulates lipid metabolism and participates in vasculitis.^[33]^ Previous research on 1-oleoyl-2-docosahexaenoyl-GPC was limited to neural regulation in the brain; the role of this metabolite in TAA has yet to be elucidated.

This study had some limitations that require consideration. First, we were unable to acquire a complete set of clinical information for all participants; consequently, we were unable to perform further stratified analysis. Second, we could only estimate the causal relationship between serum metabolites and TAA from a genetic perspective; the specific molecular mechanisms involved have yet to be verified experimentally. Third, we did not investigate differences in the serum levels of metabolites between healthy controls and patients with TAA; this needs further investigation in a large cohort.

## 5. Conclusion

Our study provides new evidence for the causal relationship between metabolites and the risk of TAA and further highlights the important role of lipid metabolism disorders in the pathogenesis of TAA.

## Author contributions

**Conceptualization:** Xiaoshan Tong.

**Data curation:** Xiaoshan Tong.

**Investigation:** Xiaoshan Tong.

**Methodology:** Yu Cui.

**Resources:** Yu Cui.

**Supervision:** Yu Cui.

**Validation:** Yu Cui.

**Writing – original draft:** Xiaoshan Tong.

**Writing – review & editing:** Yu Cui.

## Supplementary Material


