# Supplementary material for: Mendelian randomization analysis of the causal relationship between serum metabolites and thoracic aortic aneurysm
Source: Medicine (Baltimore). 2024 Sep 13;103(37):e39686. doi: 10.1097/MD.0000000000039686 (PMC11404878; doi:10.1097/MD.0000000000039686)
Supplement: Supplementary file 1 [file medi-103-e39686-s001.docx]

**Table S1 MR results of differential metabolites exhibiting causal relationships related to TAA.**

| **chr.exposure** | **pos.exposure** | **effect_allele.exposure** | **other_allele.exposure** | **eaf.exposure** | **beta.exposure** | **se.exposure** | **pval.exposure** | **SNP** | **samplesize.exposure** | **id.exposure** | **sd** | **R2** | **F** |
| --- | --- | --- | --- | --- | --- | --- | --- | --- | --- | --- | --- | --- | --- |
| 1 | 119392884 | T | A | 0.592917 | 0.174872 | 0.0149927 | 1.95E-31 | rs10802092 | 8208 | GCST90199736 | 1.3583088 | 0.01630829 | 136.044457 |
| 4 | 88304019 | G | A | 0.530035 | 0.0938716 | 0.0148331 | 2.48E-10 | rs10018448 | 8208 | GCST90199736 | 1.3438494 | 0.00485689 | 40.050191 |
| 11 | 18322517 | C | T | 0.539467 | -0.258766 | 0.01431 | 4.35E-73 | rs3802967 | 8208 | GCST90199736 | 1.2964576 | 0.03832074 | 326.990517 |
| 12 | 120986153 | T | C | 0.618461 | -0.116179 | 0.0152288 | 2.37E-14 | rs2393791 | 8208 | GCST90199736 | 1.379699 | 0.00704244 | 58.2001229 |
| 7 | 76117091 | C | T | 0.20472 | 0.112834 | 0.018756 | 1.79E-09 | rs10255591 | 8223 | GCST90199805 | 1.7008083 | 0.00438296 | 36.1909114 |
| 12 | 21161802 | A | G | 0.186831 | -0.106417 | 0.0194251 | 4.29E-08 | rs11045806 | 8223 | GCST90199805 | 1.7614828 | 0.00363738 | 30.0120386 |
| 12 | 21178615 | C | T | 0.15555 | 0.220966 | 0.0207748 | 2.02E-26 | rs4149056 | 8223 | GCST90199805 | 1.8838746 | 0.01357428 | 113.129848 |
| 19 | 15871011 | A | G | 0.29695 | -0.161683 | 0.0165605 | 1.62E-22 | rs55744319 | 8223 | GCST90199805 | 1.5017187 | 0.01146174 | 95.3195158 |
| 4 | 68489991 | C | T | 0.615895 | -0.270511 | 0.0182842 | 1.58E-49 | rs62317882 | 5524 | GCST90199850 | 1.3589479 | 0.03812756 | 218.885974 |
| 4 | 68501356 | C | T | 0.67968 | 0.162951 | 0.0190161 | 1.04E-17 | rs2603152 | 5524 | GCST90199850 | 1.4133453 | 0.01312314 | 73.4295987 |
| 5 | 22900591 | T | C | 0.006667 | -0.676314 | 0.108564 | 4.67E-10 | rs182074319 | 5524 | GCST90199850 | 8.0688691 | 0.0069789 | 38.8083414 |
| 6 | 25818527 | A | G | 0.242251 | 0.137653 | 0.0208152 | 3.76E-11 | rs9461218 | 5524 | GCST90199850 | 1.5470609 | 0.00785756 | 43.7330922 |
| 7 | 99477415 | C | T | 0.02033 | -0.697993 | 0.0615151 | 7.70E-30 | rs148982377 | 5524 | GCST90199850 | 4.5720247 | 0.02278415 | 128.747459 |
| 10 | 5197995 | T | A | 0.138874 | -0.228938 | 0.0260104 | 1.35E-18 | rs7070862 | 5524 | GCST90199850 | 1.933187 | 0.01383549 | 77.471454 |
| 7 | 76159946 | C | T | 0.298734 | 0.0914331 | 0.0162682 | 1.91E-08 | rs1859792 | 8245 | GCST90199970 | 1.4771848 | 0.00381752 | 31.5884185 |
| 12 | 21162039 | T | A | 0.714241 | 0.195248 | 0.0165847 | 5.39E-32 | rs4762697 | 8245 | GCST90199970 | 1.5059236 | 0.01653602 | 138.598314 |
| 12 | 21178615 | C | T | 0.155863 | 0.564187 | 0.0195475 | 3.55E-183 | rs4149056 | 8245 | GCST90199970 | 1.7749518 | 0.09178411 | 833.035878 |
| 19 | 15873592 | G | C | 0.295821 | -0.150725 | 0.0162078 | 1.41E-20 | rs79400241 | 8245 | GCST90199970 | 1.4717004 | 0.01038256 | 86.4813474 |
| 4 | 186204370 | T | C | 0.815573 | 0.213838 | 0.0191665 | 6.63E-29 | rs4997685 | 8049 | GCST90199992 | 1.7195459 | 0.01523294 | 124.475575 |
| 4 | 186211877 | T | A | 0.47656 | 0.119333 | 0.014961 | 1.51E-15 | rs1053094 | 8049 | GCST90199992 | 1.3422444 | 0.00784415 | 63.6209093 |
| 7 | 76159946 | C | T | 0.30076 | 0.0985258 | 0.0163329 | 1.62E-09 | rs1859792 | 8049 | GCST90199992 | 1.465326 | 0.00450173 | 36.3892692 |
| 12 | 21162039 | T | A | 0.716306 | 0.198496 | 0.0167178 | 1.63E-32 | rs4762697 | 8049 | GCST90199992 | 1.4998578 | 0.01721745 | 140.976027 |
| 12 | 21178615 | C | T | 0.158107 | 0.524674 | 0.0196894 | 1.91E-156 | rs4149056 | 8049 | GCST90199992 | 1.7664585 | 0.08108756 | 710.091188 |
| 14 | 73559459 | A | G | 0.224815 | -0.126732 | 0.0181225 | 2.69E-12 | rs72738356 | 8049 | GCST90199992 | 1.6258822 | 0.00604048 | 48.9030967 |
| 19 | 15879621 | T | C | 0.296554 | -0.177705 | 0.0162276 | 6.59E-28 | rs2108622 | 8049 | GCST90199992 | 1.4558789 | 0.0146836 | 119.919748 |
| 12 | 21162039 | T | A | 0.714576 | 0.206164 | 0.0173345 | 1.28E-32 | rs4762697 | 8066 | GCST90200026 | 1.5568273 | 0.01723855 | 141.450024 |
| 12 | 21178615 | C | T | 0.155769 | 0.522597 | 0.0206324 | 1.53E-141 | rs4149056 | 8066 | GCST90200026 | 1.8530147 | 0.07369497 | 641.555639 |
| 13 | 103065958 | T | C | 0.04717 | 0.385342 | 0.0368116 | 1.21E-25 | rs55971546 | 8066 | GCST90200026 | 3.3060834 | 0.01340637 | 109.577984 |
| 17 | 30310155 | G | A | 0.387093 | 0.0903416 | 0.0161368 | 2.16E-08 | rs11656408 | 8066 | GCST90200026 | 1.4492607 | 0.00387173 | 31.3430116 |
| 19 | 47873738 | C | T | 0.838906 | 0.250753 | 0.0211885 | 2.59E-32 | rs212100 | 8066 | GCST90200026 | 1.9029585 | 0.01707118 | 140.052817 |
| 6 | 10984097 | A | G | 0.430426 | -0.136946 | 0.0148725 | 3.32E-20 | rs9379992 | 8257 | GCST90200046 | 1.3514349 | 0.0101666 | 84.7872888 |
| 11 | 61802358 | T | C | 0.338303 | -0.247473 | 0.0154875 | 1.79E-57 | rs174546 | 8257 | GCST90200046 | 1.4073187 | 0.03000177 | 255.324804 |
| 17 | 17536144 | T | C | 0.536383 | -0.0883803 | 0.0149412 | 3.31E-09 | rs750546 | 8257 | GCST90200046 | 1.3576775 | 0.00422072 | 34.9896811 |
| 2 | 27530004 | T | C | 0.544681 | -0.0819497 | 0.0149682 | 4.38E-08 | rs6753534 | 8262 | GCST90200063 | 1.3605427 | 0.00361578 | 29.9747508 |
| 7 | 73471480 | A | G | 0.188984 | -0.105613 | 0.0190299 | 2.86E-08 | rs6976930 | 8262 | GCST90200063 | 1.7297331 | 0.00371506 | 30.8007822 |
| 11 | 116778201 | C | G | 0.868974 | -0.153691 | 0.0220238 | 2.99E-12 | rs964184 | 8262 | GCST90200063 | 2.0018653 | 0.0058611 | 48.6981389 |
| 15 | 58284964 | A | G | 0.209177 | -0.16693 | 0.018356 | 9.55E-20 | rs10162642 | 8262 | GCST90200063 | 1.6684786 | 0.00991302 | 82.7013671 |
| 15 | 58387979 | C | T | 0.344204 | 0.402273 | 0.0151253 | 7.56E-156 | rs261291 | 8262 | GCST90200063 | 1.3748224 | 0.07888051 | 707.349049 |
| 15 | 58446224 | T | C | 0.696406 | -0.198284 | 0.0162744 | 3.79E-34 | rs12914626 | 8262 | GCST90200063 | 1.4792704 | 0.01765427 | 148.444931 |
| 11 | 61804006 | C | T | 0.3627 | 0.230916 | 0.0175305 | 1.27E-39 | rs174550 | 6804 | GCST90200068 | 1.4460272 | 0.02487388 | 173.507977 |
| 15 | 58388439 | C | T | 0.385248 | 0.115854 | 0.017582 | 4.42E-11 | rs2414577 | 6804 | GCST90200068 | 1.4502752 | 0.00634287 | 43.4195696 |
| 15 | 58449185 | G | C | 0.7579 | -0.115441 | 0.0206208 | 2.17E-08 | rs572410 | 6804 | GCST90200068 | 1.7009348 | 0.00458644 | 31.3407323 |
| 6 | 10978785 | C | T | 0.451381 | -0.103813 | 0.0147669 | 2.06E-12 | rs66464172 | 8267 | GCST90200081 | 1.3426515 | 0.00594419 | 49.422512 |
| 11 | 75743976 | A | C | 0.161499 | -0.117253 | 0.0198957 | 3.78E-09 | rs499974 | 8267 | GCST90200081 | 1.8089776 | 0.00418471 | 34.7319749 |
| 11 | 116778201 | C | G | 0.86875 | -0.119699 | 0.0218633 | 4.38E-08 | rs964184 | 8267 | GCST90200081 | 1.9878778 | 0.00361355 | 29.9743391 |
| 17 | 17574533 | T | C | 0.526591 | -0.0926168 | 0.0146081 | 2.30E-10 | rs11871667 | 8267 | GCST90200081 | 1.3282129 | 0.00483996 | 40.1968552 |
| 19 | 19635342 | T | G | 0.078535 | -0.165954 | 0.0276051 | 1.84E-09 | rs2304128 | 8267 | GCST90200081 | 2.5099397 | 0.00435371 | 36.14072 |
| 11 | 61822009 | G | A | 0.30419 | -0.328142 | 0.0169823 | 3.47E-83 | rs28456 | 7731 | GCST90200103 | 1.4931875 | 0.04608069 | 373.362438 |
| 19 | 19499787 | T | C | 0.081916 | -0.212829 | 0.0293598 | 4.20E-13 | rs3794991 | 7731 | GCST90200103 | 2.5814929 | 0.00675289 | 52.5479071 |
| 19 | 44910319 | T | C | 0.199508 | 0.200541 | 0.0198647 | 5.79E-24 | rs75627662 | 7731 | GCST90200103 | 1.7466257 | 0.01301457 | 101.915991 |
| 5 | 31674119 | G | A | 0.086379 | 0.175082 | 0.0281955 | 5.31E-10 | rs1651059 | 7744 | GCST90200109 | 2.481204 | 0.00495579 | 38.55879 |
| 11 | 61820833 | G | A | 0.338294 | -0.326948 | 0.0164738 | 1.18E-87 | rs174564 | 7744 | GCST90200109 | 1.4496944 | 0.04841333 | 393.885317 |
| 19 | 19268740 | T | C | 0.074551 | -0.209338 | 0.0304765 | 6.47E-12 | rs58542926 | 7744 | GCST90200109 | 2.681932 | 0.00605723 | 47.1808718 |
| 19 | 44912921 | T | G | 0.224952 | 0.181667 | 0.0189232 | 7.98E-22 | rs483082 | 7744 | GCST90200109 | 1.6652416 | 0.01176441 | 92.1643427 |
| 4 | 82641160 | G | A | 0.192109 | 0.191197 | 0.0208226 | 4.22E-20 | rs6824723 | 7368 | GCST90200127 | 1.7873507 | 0.01131665 | 84.3125519 |
| 8 | 86468885 | G | A | 0.269226 | 0.169399 | 0.0181917 | 1.26E-20 | rs12544463 | 7368 | GCST90200127 | 1.561522 | 0.01163485 | 86.7111835 |
| 15 | 85570549 | C | A | 0.026305 | 0.287751 | 0.0516365 | 2.51E-08 | rs75062282 | 7368 | GCST90200127 | 4.4323251 | 0.00419818 | 31.0541816 |
| 12 | 21162039 | T | A | 0.714179 | 0.200516 | 0.0167302 | 4.25E-33 | rs4762697 | 8267 | GCST90200165 | 1.5211607 | 0.01708322 | 143.646748 |
| 12 | 21178615 | C | T | 0.155145 | 0.479794 | 0.0200349 | 9.73E-127 | rs4149056 | 8267 | GCST90200165 | 1.8216341 | 0.06488683 | 573.502436 |
| 19 | 15872675 | A | T | 0.296127 | -0.163201 | 0.0163224 | 1.55E-23 | rs62107766 | 8267 | GCST90200165 | 1.4840823 | 0.01195124 | 99.9718199 |
| 12 | 21162039 | T | A | 0.714615 | 0.235804 | 0.0168823 | 2.46E-44 | rs4762697 | 8231 | GCST90200170 | 1.5316443 | 0.0231588 | 195.091842 |
| 12 | 21178615 | C | T | 0.155441 | 0.557749 | 0.0200331 | 1.37E-170 | rs4149056 | 8231 | GCST90200170 | 1.8175002 | 0.08608727 | 775.142024 |
| 19 | 15665520 | T | C | 0.013588 | 0.38976 | 0.0660181 | 3.55E-09 | rs147509526 | 8231 | GCST90200170 | 5.9894828 | 0.0042178 | 34.8552732 |
| 19 | 15879621 | T | C | 0.296643 | -0.168743 | 0.0164704 | 1.24E-24 | rs2108622 | 8231 | GCST90200170 | 1.4942747 | 0.01259481 | 104.964693 |
| 2 | 233672032 | C | T | 0.014656 | 0.617646 | 0.0631631 | 1.39E-22 | rs72551330 | 8234 | GCST90200172 | 5.7315075 | 0.01148237 | 95.6208044 |
| 7 | 76164087 | C | T | 0.206495 | -0.110698 | 0.0187452 | 3.52E-09 | rs55671363 | 8234 | GCST90200172 | 1.7009655 | 0.0042185 | 34.8738096 |
| 10 | 95041116 | C | T | 0.282341 | 0.201407 | 0.0168568 | 6.64E-33 | rs1341164 | 8234 | GCST90200172 | 1.5296095 | 0.01704616 | 142.757473 |
| 10 | 99849162 | A | G | 0.059711 | 0.201183 | 0.0322895 | 4.65E-10 | rs113513789 | 8234 | GCST90200172 | 2.9299941 | 0.00469366 | 38.8203923 |
| 2 | 233713415 | A | C | 0.084712 | -0.192982 | 0.0228515 | 3.04E-17 | rs3755320 | 8233 | GCST90200241 | 2.073451 | 0.00859023 | 71.3188251 |
| 2 | 233751021 | G | C | 0.058924 | 0.151331 | 0.0272419 | 2.78E-08 | rs74787288 | 8233 | GCST90200241 | 2.4718178 | 0.00373511 | 30.8589408 |
| 7 | 64464647 | C | T | 0.696748 | -0.0788992 | 0.0141053 | 2.22E-08 | rs10252509 | 8233 | GCST90200241 | 1.2798568 | 0.00378687 | 31.2881975 |
| 12 | 21178615 | C | T | 0.155787 | 0.0966057 | 0.0176714 | 4.58E-08 | rs4149056 | 8233 | GCST90200241 | 1.60343 | 0.00361774 | 29.8857107 |
| 16 | 81527991 | C | G | 0.06893 | 0.141462 | 0.025448 | 2.72E-08 | rs7196272 | 8233 | GCST90200241 | 2.3090467 | 0.00374018 | 30.9009816 |
| 19 | 47873738 | C | T | 0.838528 | 0.256302 | 0.0174292 | 5.95E-49 | rs212100 | 8233 | GCST90200241 | 1.5814538 | 0.02559964 | 216.246498 |
| 10 | 99836239 | A | T | 0.056603 | -0.286615 | 0.0352469 | 4.24E-16 | rs17222723 | 7438 | GCST90200250 | 3.039828 | 0.00881397 | 66.1235221 |
| 12 | 21161802 | A | G | 0.182053 | -0.183065 | 0.0208111 | 1.41E-18 | rs11045806 | 7438 | GCST90200250 | 1.7948292 | 0.01029877 | 77.3785465 |
| 12 | 21178615 | C | T | 0.160813 | 0.406891 | 0.0215012 | 7.22E-80 | rs4149056 | 7438 | GCST90200250 | 1.8543461 | 0.04594768 | 358.121809 |
| 19 | 47857434 | A | T | 0.156727 | -0.219784 | 0.022263 | 5.50E-23 | rs76531193 | 7438 | GCST90200250 | 1.9200466 | 0.0129369 | 97.4596335 |
| 6 | 25762418 | T | C | 0.430149 | 0.112088 | 0.0163168 | 6.44E-12 | rs3799340 | 7512 | GCST90200253 | 1.4142063 | 0.00624436 | 47.1898134 |
| 6 | 100635818 | T | C | 0.148596 | -0.126919 | 0.0231726 | 4.32E-08 | rs34199055 | 7512 | GCST90200253 | 2.0084108 | 0.00397862 | 29.9987911 |
| 14 | 80796448 | C | A | 0.012327 | 0.485674 | 0.0735864 | 4.11E-11 | rs114538296 | 7512 | GCST90200253 | 6.3778654 | 0.00576691 | 43.5606745 |
| 19 | 32791891 | A | G | 0.191175 | 0.158043 | 0.0204763 | 1.18E-14 | rs8106748 | 7512 | GCST90200253 | 1.7747177 | 0.00787003 | 59.5727382 |
| 2 | 43845437 | T | G | 0.685493 | 0.0983145 | 0.0164331 | 2.19E-09 | rs4299376 | 8203 | GCST90200257 | 1.4883527 | 0.00434548 | 35.7928396 |
| 7 | 99543750 | A | G | 0.049265 | -0.408715 | 0.0349303 | 1.26E-31 | rs10278040 | 8203 | GCST90200257 | 3.1636518 | 0.01642023 | 136.910426 |
| 7 | 99672916 | C | T | 0.936493 | 0.929459 | 0.029496 | 6.07E-218 | rs776746 | 8203 | GCST90200257 | 2.6714649 | 0.10800189 | 992.96565 |
| 7 | 99676693 | G | A | 0.052274 | 0.28152 | 0.0344371 | 2.96E-16 | rs62471929 | 8203 | GCST90200257 | 3.1189824 | 0.00808302 | 66.8290618 |
| 10 | 94909603 | C | T | 0.196107 | 0.173928 | 0.0192353 | 1.54E-19 | rs57991629 | 8203 | GCST90200257 | 1.7421491 | 0.00987111 | 81.7600367 |
| 12 | 21162039 | T | A | 0.714464 | 0.178699 | 0.0169938 | 7.33E-26 | rs4762697 | 8203 | GCST90200257 | 1.5391355 | 0.01330393 | 110.576602 |
| 12 | 21178615 | C | T | 0.15654 | 0.456077 | 0.0203329 | 1.98E-111 | rs4149056 | 8203 | GCST90200257 | 1.8415592 | 0.05780328 | 503.12708 |
| 19 | 47865277 | T | C | 0.839218 | 0.159946 | 0.0208886 | 1.90E-14 | rs296391 | 8203 | GCST90200257 | 1.8918892 | 0.00709851 | 58.6311078 |
| 3 | 52078217 | A | C | 0.010632 | 0.421325 | 0.0717454 | 4.29E-09 | rs148804382 | 8235 | GCST90200343 | 6.5106727 | 0.00417131 | 34.4862789 |
| 9 | 127715489 | A | G | 0.510457 | -0.167923 | 0.0144172 | 2.37E-31 | rs504434 | 8235 | GCST90200343 | 1.3083162 | 0.01621073 | 135.662096 |
| 16 | 20346926 | C | A | 0.179504 | -0.112808 | 0.0191073 | 3.55E-09 | rs9928003 | 8235 | GCST90200343 | 1.7339283 | 0.00421588 | 34.8562895 |
| 5 | 132341354 | T | G | 0.476425 | -0.139313 | 0.0151713 | 4.20E-20 | rs11242109 | 8225 | GCST90200471 | 1.3759123 | 0.01015025 | 84.3213783 |
| 5 | 151343594 | A | C | 0.011987 | -0.435487 | 0.0697394 | 4.25E-10 | rs77010315 | 8225 | GCST90200471 | 6.3247907 | 0.00471964 | 38.9936579 |
| 6 | 160122116 | T | C | 0.068507 | -0.32469 | 0.029745 | 9.69E-28 | rs12208357 | 8225 | GCST90200471 | 2.6976272 | 0.0142834 | 119.154344 |
| 6 | 160154805 | A | G | 0.02525 | -0.364144 | 0.0481867 | 4.13E-14 | rs34059508 | 8225 | GCST90200471 | 4.3701379 | 0.00689693 | 57.1073419 |
| 10 | 59707424 | T | G | 0.769265 | 0.178377 | 0.0183417 | 2.35E-22 | rs1171617 | 8225 | GCST90200471 | 1.6634415 | 0.01137108 | 94.5798323 |
| 6 | 25798704 | A | G | 0.544739 | -0.258319 | 0.0158671 | 1.37E-59 | rs1165215 | 7071 | GCST90200537 | 1.3342524 | 0.03613883 | 265.04376 |
| 10 | 95060813 | A | G | 0.292737 | 0.174229 | 0.0175423 | 3.02E-23 | rs77794699 | 7071 | GCST90200537 | 1.4751186 | 0.01376231 | 98.6433517 |
| 12 | 20944395 | G | A | 0.226167 | 0.109375 | 0.0192102 | 1.24E-08 | rs12814270 | 7071 | GCST90200537 | 1.6153711 | 0.00456486 | 32.4169655 |
| 17 | 50683692 | A | G | 0.068758 | -0.195702 | 0.0316994 | 6.67E-10 | rs11568591 | 7071 | GCST90200537 | 2.6655784 | 0.00536284 | 38.1143435 |
| 18 | 58814558 | G | A | 0.022285 | 0.302925 | 0.0542577 | 2.36E-08 | rs73437925 | 7071 | GCST90200537 | 4.5624887 | 0.00439014 | 31.1707659 |
| 10 | 99844724 | C | T | 0.057249 | -0.580097 | 0.0327696 | 4.02E-70 | rs72838129 | 7982 | GCST90200586 | 2.9277029 | 0.03778573 | 313.371112 |
| 10 | 100126891 | C | T | 0.016998 | 0.464597 | 0.0602426 | 1.24E-14 | rs189017081 | 7982 | GCST90200586 | 5.3821967 | 0.00739806 | 59.4764978 |
| 12 | 21162039 | T | A | 0.716039 | 0.169972 | 0.017203 | 5.06E-23 | rs4762697 | 7982 | GCST90200586 | 1.5369511 | 0.01208545 | 97.6217055 |
| 12 | 21215863 | A | T | 0.168675 | 0.351432 | 0.0201586 | 4.60E-68 | rs2900478 | 7982 | GCST90200586 | 1.8010104 | 0.03668816 | 303.921815 |
| 19 | 47873738 | C | T | 0.841223 | 0.331612 | 0.0210966 | 1.13E-55 | rs212100 | 7982 | GCST90200586 | 1.8848133 | 0.03003239 | 247.078832 |
| 6 | 160065899 | T | C | 0.124676 | 0.165053 | 0.0227055 | 3.61E-13 | rs671439 | 8241 | GCST90200601 | 2.0612042 | 0.00637285 | 52.8426641 |
| 6 | 160130454 | T | C | 0.069126 | 0.219811 | 0.0292217 | 5.39E-14 | rs112201728 | 8241 | GCST90200601 | 2.6527446 | 0.00682089 | 56.58325 |
| 11 | 27090577 | A | G | 0.012607 | 0.783458 | 0.0660974 | 2.07E-32 | rs142059872 | 8241 | GCST90200601 | 6.0003189 | 0.01676659 | 140.495588 |
| 11 | 27125577 | C | T | 0.039363 | -0.526832 | 0.0379546 | 8.31E-44 | rs2304913 | 8241 | GCST90200601 | 3.4455168 | 0.02285082 | 192.670602 |
| 11 | 27631832 | T | G | 0.005591 | -0.551225 | 0.0989853 | 2.57E-08 | rs188956377 | 8241 | GCST90200601 | 8.985881 | 0.00374982 | 31.0110453 |
| 10 | 95042415 | T | A | 0.124118 | 0.199025 | 0.0249157 | 1.37E-15 | rs11572168 | 6391 | GCST90200630 | 1.991854 | 0.00988827 | 63.8071106 |
| 12 | 21162039 | T | A | 0.724334 | 0.127276 | 0.0183799 | 4.37E-12 | rs4762697 | 6391 | GCST90200630 | 1.4693578 | 0.00744949 | 47.9520024 |
| 12 | 21178615 | C | T | 0.169225 | 0.379803 | 0.0210749 | 1.32E-72 | rs4149056 | 6391 | GCST90200630 | 1.6848061 | 0.04837476 | 324.777352 |
| 19 | 47873738 | C | T | 0.84717 | 0.160783 | 0.0227913 | 1.73E-12 | rs212100 | 6391 | GCST90200630 | 1.8220215 | 0.00772929 | 49.7670686 |
| 4 | 179600521 | A | T | 0.243766 | -0.104192 | 0.0179893 | 6.96E-09 | rs62333059 | 8167 | GCST90200664 | 1.6257192 | 0.0040917 | 33.5459597 |
| 10 | 98391509 | G | A | 0.358392 | -0.104477 | 0.0158403 | 4.23E-11 | rs10786415 | 8167 | GCST90200664 | 1.431511 | 0.00529969 | 43.5025367 |
| 11 | 69430283 | T | C | 0.020523 | 0.323203 | 0.0533403 | 1.37E-09 | rs142171054 | 8167 | GCST90200664 | 4.8204406 | 0.00447647 | 36.7146894 |
| 17 | 77756770 | G | C | 0.01529 | 0.344913 | 0.0620602 | 2.73E-08 | rs117250991 | 8167 | GCST90200664 | 5.6084706 | 0.00376875 | 30.8882131 |
| 4 | 69103961 | A | C | 0.459862 | -0.120089 | 0.0156006 | 1.39E-14 | rs7375178 | 8002 | GCST90200670 | 1.3955345 | 0.0073524 | 59.2548432 |
| 12 | 21161802 | A | G | 0.183841 | -0.121363 | 0.0199163 | 1.10E-09 | rs11045806 | 8002 | GCST90200670 | 1.7815907 | 0.00462013 | 37.1325939 |
| 12 | 21178615 | C | T | 0.158537 | 0.310638 | 0.0208638 | 3.90E-50 | rs4149056 | 8002 | GCST90200670 | 1.8663483 | 0.02696261 | 221.677871 |
| 5 | 31674119 | G | A | 0.0868 | -0.166064 | 0.0282832 | 4.32E-09 | rs1651059 | 7649 | GCST90200902 | 2.473608 | 0.00448796 | 34.4741764 |
| 11 | 61820833 | G | A | 0.336609 | 0.309609 | 0.0166232 | 2.01E-77 | rs174564 | 7649 | GCST90200902 | 1.4538412 | 0.04339498 | 346.894883 |
| 19 | 19553268 | T | C | 0.078076 | 0.18211 | 0.0299914 | 1.26E-09 | rs17216588 | 7649 | GCST90200902 | 2.6230047 | 0.00479837 | 36.8700826 |
| 19 | 44913484 | T | C | 0.225481 | -0.178794 | 0.0189961 | 4.86E-21 | rs438811 | 7649 | GCST90200902 | 1.6613716 | 0.01145206 | 88.5884263 |
| 11 | 61822009 | G | A | 0.301605 | 0.30322 | 0.0171121 | 2.96E-70 | rs28456 | 7623 | GCST90200904 | 1.4940539 | 0.03956972 | 313.985121 |
| 19 | 19551411 | T | C | 0.077534 | 0.196909 | 0.0300635 | 5.76E-11 | rs17216525 | 7623 | GCST90200904 | 2.624838 | 0.00559761 | 42.8994825 |
| 19 | 44912921 | T | G | 0.226461 | -0.177755 | 0.0190295 | 9.54E-21 | rs483082 | 7623 | GCST90200904 | 1.6614617 | 0.01131965 | 87.2547135 |
| 11 | 61822009 | G | A | 0.30108 | 0.284671 | 0.0172101 | 1.86E-61 | rs28456 | 7617 | GCST90200907 | 1.5020188 | 0.03468324 | 273.602251 |
| 11 | 116778201 | C | G | 0.868563 | 0.13873 | 0.0237306 | 5.03E-09 | rs964184 | 7617 | GCST90200907 | 2.0710982 | 0.00446795 | 34.1761655 |
| 19 | 19268740 | T | C | 0.07356 | 0.227033 | 0.0309628 | 2.26E-13 | rs58542926 | 7617 | GCST90200907 | 2.7022916 | 0.00701087 | 53.7647364 |
| 19 | 44912921 | T | G | 0.226311 | -0.180649 | 0.0191006 | 3.15E-21 | rs483082 | 7617 | GCST90200907 | 1.667013 | 0.01161009 | 89.4493325 |
| 11 | 61790331 | C | T | 0.340013 | 0.311885 | 0.0165777 | 5.85E-79 | rs102275 | 7668 | GCST90200908 | 1.4516614 | 0.04413355 | 353.948783 |
| 19 | 19268740 | T | C | 0.073789 | 0.205034 | 0.0308024 | 2.81E-11 | rs58542926 | 7668 | GCST90200908 | 2.6972774 | 0.0057466 | 44.308052 |
| 19 | 44912921 | T | G | 0.225421 | -0.182733 | 0.018984 | 6.23E-22 | rs483082 | 7668 | GCST90200908 | 1.6623742 | 0.01194187 | 92.6527935 |
| 8 | 18085712 | G | T | 0.024692 | 0.296844 | 0.0517329 | 9.58E-09 | rs151142780 | 7388 | GCST90201020 | 4.4466226 | 0.00443795 | 32.9247815 |
| 8 | 18172570 | A | G | 0.285461 | 0.12155 | 0.0180088 | 1.48E-11 | rs4548221 | 7388 | GCST90201020 | 1.547919 | 0.00613 | 45.5554537 |
| 8 | 18348007 | C | A | 0.809485 | 0.499318 | 0.0198668 | 2.15E-139 | rs1493042 | 7388 | GCST90201020 | 1.7076205 | 0.07878613 | 631.68215 |
| 8 | 18387243 | T | C | 0.195643 | -0.746632 | 0.0184392 | 4.94065645841247e-324 | rs75501607 | 7388 | GCST90201020 | 1.5849133 | 0.18165802 | 1639.56655 |
| 19 | 40847202 | C | T | 0.576193 | -0.162491 | 0.016193 | 1.07E-23 | rs56113850 | 7388 | GCST90201020 | 1.3918446 | 0.01344974 | 100.694092 |

**MR=mendelian randomization; TAA=thoracic aortic aneurysm**
